# Multiple‐Use Microplate Assay for Submicromolar Ultra High‐Throughput Separation of Amines Based on their Degree of Substitution

**DOI:** 10.1002/open.202500491

**Published:** 2025-12-01

**Authors:** Panna Vezse, Tünde Tóth, Péter Huszthy, Ádám Golcs

**Affiliations:** ^1^ Department of Organic Chemistry and Technology Faculty of Chemical Technology and Biotechnology Budapest University of Technology and Economics Budapest Hungary; ^2^ HUN‐REN Centre for Energy Research Budapest Hungary; ^3^ Department of Pharmaceutical Chemistry Semmelweis University Budapest Hungary; ^4^ Center for Pharmacology and Drug Research & Development Semmelweis University Budapest Hungary

**Keywords:** high‐throughput, library synthesis, microplate, molecular recognition, separation

## Abstract

Small‐molecule amines, typically studied in their more stable and water‐soluble protonated forms, are of central importance in drug discovery. Their structural diversification often relies on *N*‐alkylation, yielding mixtures of analogs with varying degrees of substitution‐posing a key challenge for purification. While advanced chromatographic techniques exist, no high‐throughput, broadly applicable alternative has emerged that aligns with the capabilities of automated synthesis. Here, a reusable microplate‐based assay enabling ultra‐high‐throughput, parallel separation of protonated amines‐including alkyl‐, aryl‐, and aralkylamines‐at submicromolar levels is reported. The method exploits a covalently immobilized tris(pyridino)‐crown ether selector, which forms reversible host–guest complexes by *H*‐bonds, which differ with the degree of *N*‐substitution. This supramolecular recognition strategy eliminates the need for compound‐specific method development, derivatization, or preparative‐scale quantities. In addition, the present article introduces a generally applicable surface‐functionalization protocol for customizing standard commercial microplates into molecular recognition platforms. The present approach resolves key limitations of current separation technologies‐such as high energy use, low integration with liquid‐handling systems, inevitable sample dilution, and time‐intensive workflows‐offering a transformative tool for rapid and efficient purification directly compatible with modern synthesis pipelines.

## Graphical abstract

A reusable microplate platform for ultra‐high‐throughput, submicromolar separation of protonated amines – including alkyl‐, aryl‐, and aralkyl‐types – using a tris(pyridino)‐crown ether selector was introduced; the method enables rapid, zero‐energy, parallel purification fully compatible with automated synthesis workflows.

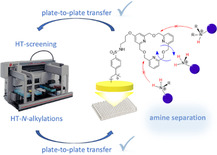

## 1 Introduction

A large number of biologically active synthetic molecules contain at least one amine group [1]. All of the main classes of amines, i.e., aromatic or aliphatic, primary, secondary, and tertiary ones, are among the top 13 most frequent functional groups occurring in bioactive molecules in medicinal chemistry literature [2]. Under physiologically relevant conditions, these compounds are usually in their protonated forms [1]. Solubility in body fluids (pH ≤ 7.4 aqueous solutions) is an essential criterion for these molecules to influence biological functions [3]. In most of the cases, it means a log*S* value preferably above −3 (in water) [4].

The modification of the structural backbone of drug‐like molecules is mainly carried out by *N*‐substitution in the cases of precursors containing amino groups. These reactions are typically not selective and require the separation of the target product from a mixture of differently substituted analogs [5]. The purification of these analogs can particularly be challenging due to similarities in chemical character, molecular size, or polarity.

Generally, the development of submicromolar amine‐separation techniques is limited to improving highly compound‐specified chromatographic methods [6, 7] or has a strong focus on enantioseparation [8, 9]. In addition to these time consuming and compound‐specific methods, a parallel or automatable tool still remains of demand in the development of chromatography‐based procedures. Similarly, the molecular imprinted polymeric approaches [10] and various extraction platforms [11, 12, 13–14] are restricted to the selective separation of predefined smaller component‐classes or to specific instrumentation. This high specificity makes generalizability and wide applicability impossible in practice.

Thus, a high‐throughput separation technique for differently substituted amines is of fundamental importance to synthetic chemists working on preliminary tests of new compounds for various applications. The new derivatives could be studied separately, even in the first step of discovery research, without optimizing synthetic procedures or preparing larger quantities of molecular candidates.

Since synthetic host molecules such as crown ethers embedded in stationary phases have already proven their efficiency in separation of protonated amines [15, 16–17], the exploitation of their well‐defined preference in molecular recognition also offers an attractive alternative approach for developing high‐throughput separation tools. Nonetheless, examples for the microplate‐based applications of synthetic hosts are quite rare and the majority of them focus on monitoring of permeability [18, 19] or receptor‐ligand interactions [20], besides the most common optochemical HTS‐platforms for ionic components [21, 22]. Thus, supramolecular interaction‐based microplate platforms mean a completely new field of separation science and purification technology.

Recently, we reported a new crown ether type selector molecule, which exhibited different affinities for primary, secondary, and tertiary ammonium ions [23]. The discrimination was realized by the formation of reversible complexes with different stabilities strengthened by the increasing number of *H*‐bonds as a function of the degree of *N*‐substitution. The immobilization of this selector molecule to the microplate provides a universal assay platform for the desired unique functions in separation and opens the door to automated implementations.

Herein, we report the development of a polystyrene (PS)‐based 96‐well microplate covalently functionalized with crown ether selector molecules on its surface for high‐throughput separation of differently substituted structurally analog ammonium salts based on their degree of *N*‐substitution. We demonstrate the utility of our tool in the ultrafast parallel separation of a great diversity of ammonium ions, including alkyl‐, aryl‐, and aralkyl type model compounds, in aqueous solutions of relevant concentrations.

## 2 Results and Discussion

### 2.1 Surface Functionalization of PS Microplates

**SCHEME 1:**
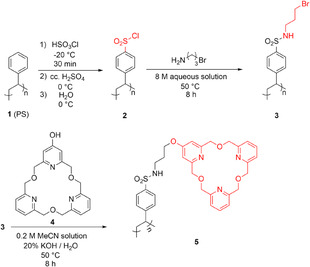
Covalent functionalization of the surface of PS microplate wells.

The chemical modification of PS surfaces of the microplate wells was carried out similarly to a previously reported method [24], which was specified for the desired purpose (detailed procedure can be found in Subsection 4.2.) in the aim of introducing reactive functional groups for immobilizing the selector molecules. This involves a two‐step wet‐chemical modification procedure. The first step is the activation of the indifferent apolar surface by creating chlorosulfonyl groups, while the second step is a reaction with a bifunctional molecular linker. Its aliphatic primary amino group serves as an anchorage to the preactivated polymer surface by sulfonamide bonds. The second function, the terminal bromide unit, can be used for immobilization of the selector molecules, which was performed by a new method (see Subsection 4.2.) under an inert atmosphere. The summary of the preparation process can be seen in Scheme 1.

**FIGURE 1:**
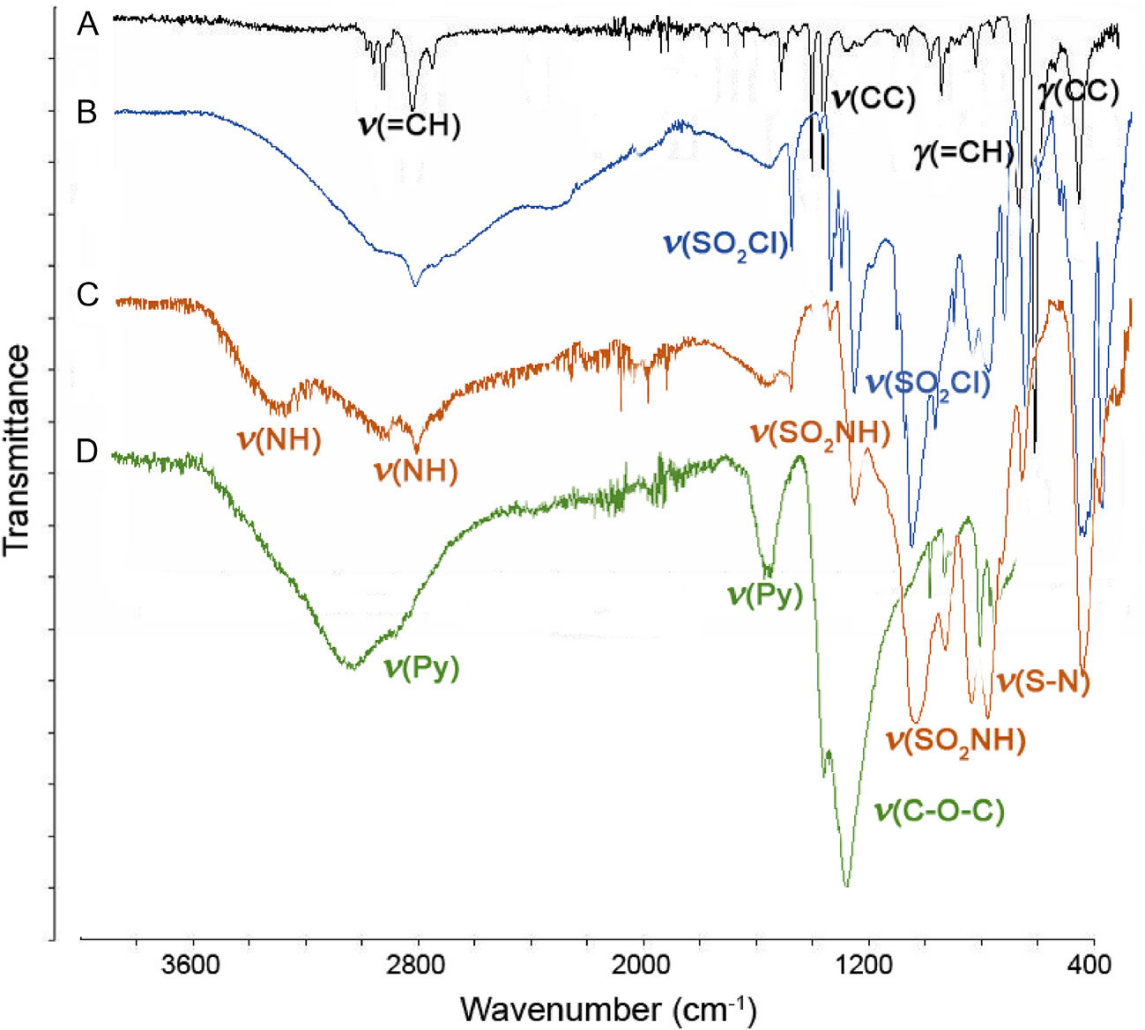
Solid phase ATR‐FTIR spectra to characterize the functionalized surface and prove the desired chemical modification after each step of the synthetic procedure (A: spectrum of the unmodified PS, B: spectrum after chloro‐sulfonylation, C: spectrum after introducing the amine functionalized molecular linker by sulfonamide bonds, and D: microplate containing the surface‐immobilized selector molecules).

After each step of the procedure, the modified surface was analyzed by solid phase attenuated total reflectance fourier‐transform infrared (ATR‐FTIR) spectroscopy. (Elemental analysis was also performed to determine the extent of labeling, but this method was not able to give valuable results as the amount of the covalently anchored compounds on the surface was not comparable to that of the bulk polymer). Results can be found in Figure 1.

Initially, the unmodified PS surface was characterized as a reference (part “A” in Figure 1). The absorption peaks at wavenumbers of 3061 and 3026 cm^−1^ referred to the aromatic C—H stretching vibration absorptions, the two other ones at 2922 and 2849 cm^−1^ indicate the presence of methylene groups. There are three absorption peaks at the wavenumbers of 1601, 1493, and 1452 cm^−1^ corresponding to the aromatic C=C stretching vibration absorptions, while the absorption peaks at wavenumbers 756 and 698 cm^−1^ correspond to C—H out‐of‐plane (deformation) bending vibration absorptions. The other vibrations at higher energy‐excitations belong to the fingerprint region and referring to the aliphatic C—C vibrations. The appearance of the chlorosulfonyl groups on the surface, accompanied by the occurrence of two characteristic peaks at 1373 and 1171 cm^−1^ due to the presence of the S=O valence bonds (part “B” in Figure 1). These peaks were shifted to 1312 and 1156 cm^−1^, respectively, when replacing the chlorosulfonyl groups by sulfonamide units (part “C” in Figure 1). In addition, the broad signals at 3369 and 3278 cm^−1^ correspond to the associated and free *NH* of the linker, respectively. (The latter one can only be eliminated by extensive washing with methanol). Spectrum “D” refers to the microplate‐surface covalently functionalized with the selector molecules. There is a wide shoulder around the 3100 cm^−1^ area, which serves as evidence for the aromatic vibrations of the pyridine ring, while peaks between 1650 and 1550 cm^−1^ correspond to the vibrations of conjugated C=C and C=N bonds. The ethereal bonds in the macrocycle ring are confirmed by strong peaks around 1000 cm^−1^ attributed to the nonequivalent C—O stretching vibrations. According to the expectations, as the structural complexity of the polymer surface increases, the other bands tend to blend together, resulting in broadened absorption ranges.

In order to give information on the extent of labeling by the immobilized selector molecules, the functionalized microplates were studied by three orthogonal methods. In the last step of the procedure, the ratio of the rest of the selector molecules, ‐ i.e., that did not bind covalently to the surface, was calculated relying on a UV/Vis calibration curve based on the Lambert–Beer equation (detailed information can be found in the Supporting Information). The loading density of the selector molecule was subsequently determined indirectly from the concentration of the initial solution used for the surface treatment. Based on this calculation, 7 ± 2% of the applied macrocycles were immobilized to the surface covalently. As the effective immobilization does not necessarily mean that all the selector molecules are in their advantageous conformational state, this calculation was extended by receptor‐binding studies. According to the previously published preliminary studies on the applied macrocycle [23], protonated benzylamine can be complexed most effectively among the reported model compounds. The logarithm of the complex stability constant of log*K* = 4.9 ± 0.3 refers to a very strong coordination among reversible host molecules. Thus, the complexation of this strongly bonded guest molecule can also be used to determine the amount of the active selector molecules on the polymer surface. Detailed study can be found in Subsection 2.3. It can be seen that the covalently attached selector molecules complex ≈75% of the guest molecules from their aqueous solutions (100 µL, 10^−2 ^M), which means that ≈6% (7.5 × 10^−7 ^mol) of the macrocycles were effectively immobilized to the surface and are present in their active form for complexation.

To further validate the surface concentration of the immobilized selector molecules, a conductometric acid‐uptake titration was performed. Due to the small total volume of the wells (350 µL), the functionalized microplate was carefully disassembled and the individual wells were detached and used separately for titration measurements.

Each well was immersed in 5.0 mL of deionized water and equilibrated for 30 min under gentle stirring to ensure full wetting of the surface. The initial conductivity (*κ*_0_) of the solution was recorded using a Mettler Toledo SevenCompact S230 conductometer equipped with an InLab 731 conductivity electrode (cell constant: 0.57 cm^−1^). Subsequently, HCl solution was added stepwise (10 µL increments, 0.01 M) under continuous stirring at 25 ± 0.5°C. The conductivity was recorded after each addition once equilibrium was reached (≈30 s). The titration curve (*κ* vs. nH^+^) exhibited a distinct break point corresponding to the stoichiometric protonation of the pyridine nitrogen atoms on the immobilized selector molecules. Blank experiments performed with nonfunctionalized wells under identical conditions showed no measurable acid uptake, confirming that the observed conductivity change originated exclusively from the protonation of the selector units. Since each selector molecule contains three pyridine groups, the total number of protonated sites obtained from the titration was divided by three to yield the amount of immobilized selector molecules. Based on three independent parallel measurements (RSD < 5%), the immobilization density was determined to be (8 ± 0.4) × 10^−7^ mol of selector molecules per plate well, which is in excellent agreement with the results obtained from UV/Vis and receptor‐binding assays. Moreover, these studies also indicated that the macrocycles preserved their molecular recognition ability in their immobilized form.

### 2.2 Separation Mechanism and Concept

**FIGURE 2:**
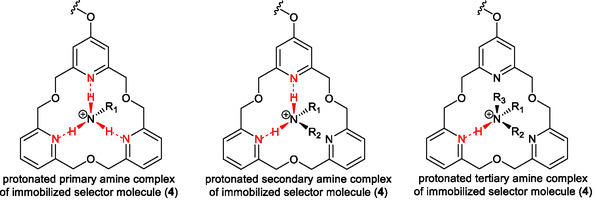
Different numbers of *H*‐bonds inside the complexes formed by the immobilized selector molecule (**4**) with protonated amines of different degrees of *N*‐substitutions.

The basic and nucleophilic pyridine‐*N* atoms of the applied selector molecule tend to coordinate electrophilic organic ammonium cations in the macrocycle ring by forming stable, but reversible, inclusion complexes. The complex is mostly stabilized by intermolecular *H*‐bonds between the nucleophilic units of the host and the ammonium (or amine) unit of the guest molecules. Since there are three symmetrically arranged pyridine‐*N* atoms with higher nucleophilicity than oxygens, primary ammonium groups of a tetrahedral structure can be fixed by three *H*‐bonds connecting to *N*‐atoms at alternating positions. The number of *H*‐bonds decreases as the degree of substitution increases, thus complexes with different stabilities are formed (Figure 2). This property can be exploited for separation.

During the separation process, aqueous solutions of the amines were added to each well of the 96‐well‐assay. After contacting the functionalized polymer surface at the bottom of the wells, a complexation equilibrium begins to be established while continuously shaking, thus the separation actually can be called a solid phase extraction. Naturally, the kinetics of the process can be strongly influenced by parameter‐optimization, but in order to reach the equilibrium‐state, incubation for a certain period of time is necessary.

Ideally, the most preferred amine analog would be present in an amount equivalent to the selector molecules, allowing a perfect 1:1 complexation. However, this is not feasible in practice. In our experiments, the total number of dissolved amines was deliberately kept below the number of active binding sites on the microplate surface, ensuring that competition among structural analogs for binding sites took place in all cases. This setup realistically reflects the conditions anticipated in practical applications, where the method is intended to support high‐throughput scaffold synthesis directly on microplate platforms.

**FIGURE 3:**
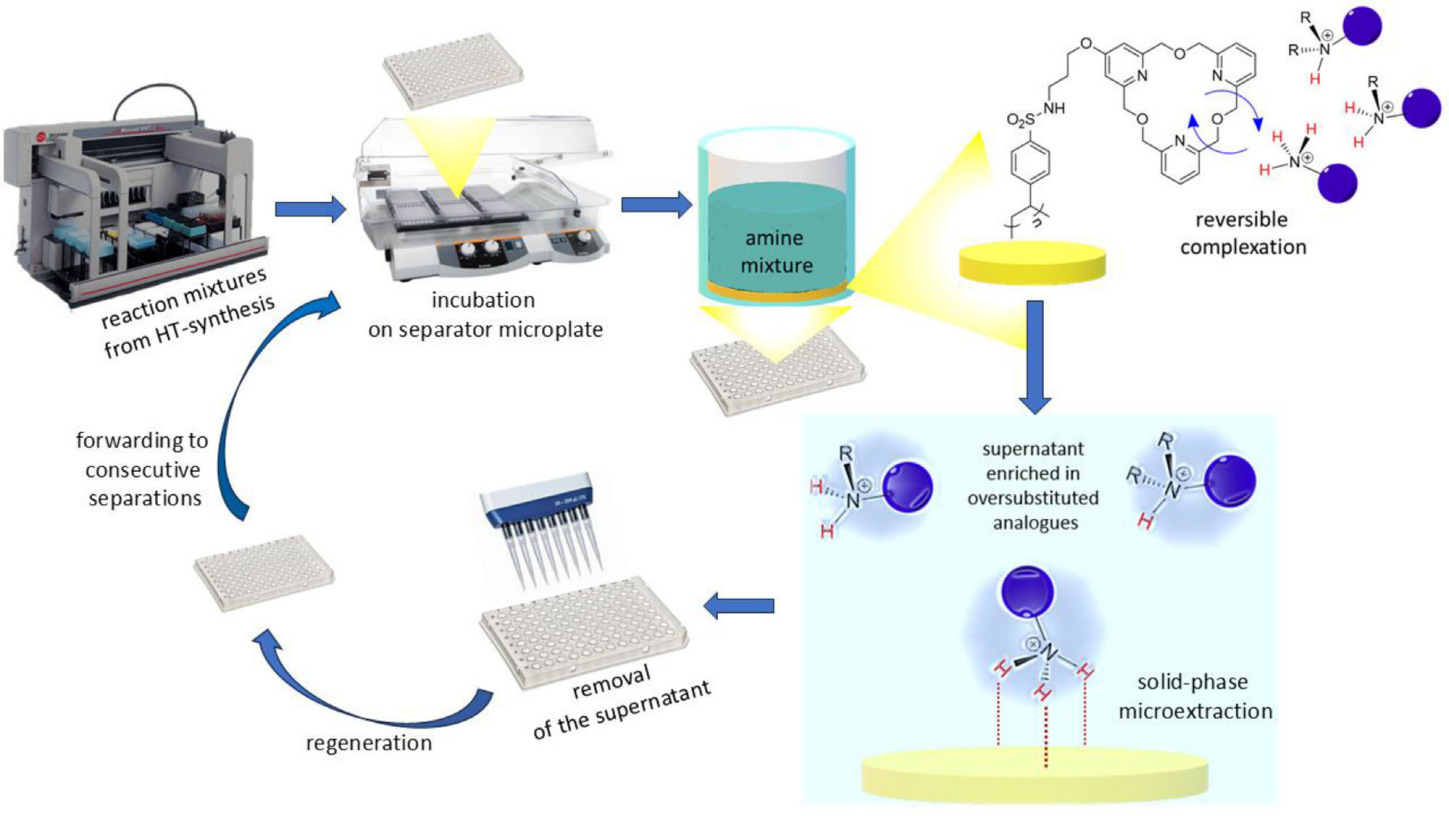
Concept of the novel high‐throughput purification method and the applied automated instrumental background (liquid handling workstation and programable plate incubator).

After a proper incubation time, the aqueous phases are enriched in the amines of higher degrees of *N*‐substitution as their complexation are less preferred by the immobilized selector molecules. Although the discriminative power of the selector molecules manifests even in the equilibrium‐based separations, it is recommended to interrupt these separations at the initial stage of the process, before the competition attributed to the complexation of the thermodynamically less preferred amines is strongly expressed (selectivity is provided by both kinetic and thermodynamic stability relations). Schematic representation of the separation process can be seen in Figure 3.

The liquid phases are removed with multichannel pipettes at the end of the separations. If the purity is not appropriate, the separation steps can be repeated several times. This is an extremely simple and rapid separation method requiring only a direct plate‐to‐plate solution transfer through an automated workflow. The only limitation–which is also a criterion for multiple use and regenerability–is the reversible binding property of the selector molecules. This feature hinders the complete recovery of the liquid phase after the separation step, because the suction effect at the bottom of the wells generates an intense flow when removing the entire amount of solution, leading to partial decomplexation. Due to these observations, only half of the applied liquid phase was recovered upon incubation at the expense of material loss.

### 2.3 Studies on Separability of Amines Based on their Degree of *N*‐Substitution

**FIGURE 4:**
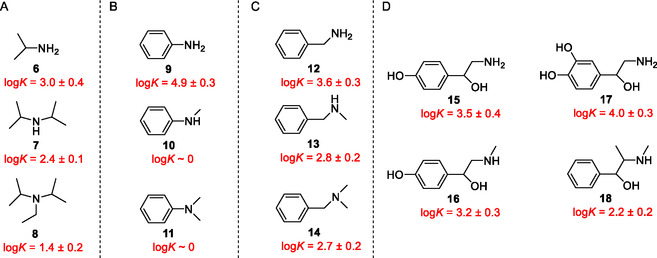
The classes of guest molecules including (A) aliphatic, (B) aromatic, (C) aralkyl, and (D) bioactive amines as model compounds and the logarithms of their previously determined complex stability constants (log*K* in acetonitrile for amines as hydrochloride salts) referring to the substitution‐degree‐based separation ability of host molecule **4**.

Studies on separability were carried out by measuring the concentrations of the solutions in the wells as a function of the incubation time. In these cases, model compounds were studied separately using their single‐component aqueous solutions, while the obtained results were expressed as the percentage ratio of the reversibly bonded protonated amine on the bottom of the well related to the full amount of the amine in the initial source solution. The same model amines were applied as in our preliminary study [23]. These model amines (**6**–**18**) and the logarithms of the stability constants of their complexes [23] are summarized in Figure 4.

All the structural classes of the selected model amines contained three analogs of different degrees of *N*‐substitution extended by some bioactive small‐molecule amines (see group “D” in Figure 4), i.e., octopamine (**15**), synephrine (**16**), norepinephrine (**17**), and ephedrine (**18**) containing additional substituents in both aliphatic and aromatic units. Since coordination takes place between the macrocycle cavity and the amine (protonated amine at pH = 7.0 in aqueous solutions) function of the guest molecule, aralkyl amines with an aromatic unit more than three *C*‐atoms away from the complexed ammonium groups can be regarded as alkylamines in the context of separation, as the influence of aromatic *π–* *π* interactions is negligible in these cases.

**FIGURE 5:**
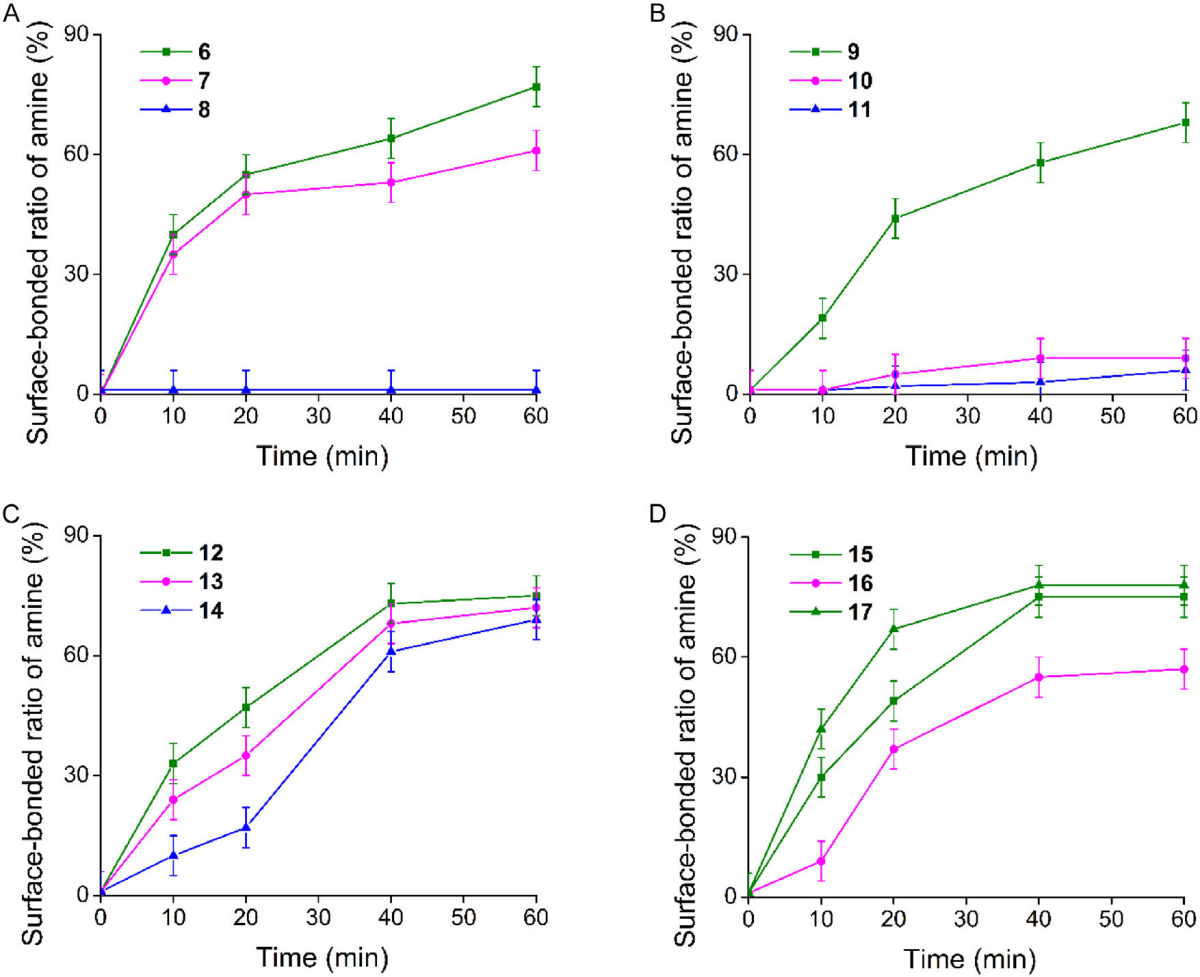
Kinetic studies on separability of different amine classes ((A) aliphatic, (B) aromatic, (C) aralkyl, and (D) some bioactive amines) as model compounds; the diagrams were obtained by measuring the ratio of the surface‐adsorbed amine from single‐component solutions; the curves for primary amines are uniformly green, while those for secondary and tertiary ones are magenta and blue, respectively).

The separability of alkyl derivatives (group “A” in Figure 4) was investigated first. Preliminary studies [23] showed a 4‐ and 10‐fold differences in complex stabilities of primary over secondary and tertiary derivatives, respectively. These relations were also reflected in the results, which can be seen in part “A” of Figure 5.

The curves obtained for primary and secondary derivatives predict a limited effectiveness in their separation from mixtures (see part “A” in Figure 5). However, the tertiary ones can be easily separated since they showed almost no affinity to the host molecules. Although preliminary studies indicating a weak intermolecular interaction, it is only due to the presence of the positive charge of the protonated *Hünig*'s base, which is too sterically shielded to form effective interactions.

According to the experiments, 60 min of incubation time was required to reach the bonding equilibria. In general, differences in complex stabilities between distinct compound classes did not significantly affect either the kinetic (i.e., time‐dependent) or thermodynamic (i.e., equilibrium‐state binding efficiency) behavior of the system. However, within individual compound classes, variations in stability constants were clearly reflected in the measured results. Accordingly, the calculated stability constants demonstrated strong predictive power for estimating relative binding preferences among closely related analogs.

The next group is the aromatic amines (see part “B” in Figure 5), where only the primary derivative could be bonded effectively; the complexation of the highly substituted ones was negligible, probably attributed to their highly shielded protonated amine centers.

The aralkyl analogs (see part “C” in Figure 5) have an increased conformational flexibility compared to the former group, thus the intermolecular *π–* *π* interactions gain a more important role inside the complex due to the more effective overlapping of the aromatic units. This can also make the more substituted derivatives significantly bonded. Thus, the expected separability is less effective in this case, which is also supported by the complex stability constants (Figure 4).

Among the last compound group (see part “D” in Figure 5), the influence of the different aromatic substituents proved to be very weak. Connected to the aromatic moieties, the steric effects are expected to be weak and from the point of view of stereoelectronic interactions, a large difference is expected only for substituents of significantly different electronic properties. Thus, the complexation of synephrine (**16**) is weaker compared to the other aralkyl analogs.

In summary, the obtained experimental results showed a very good correlation with the complex stability constants. The competition between secondary and tertiary amines is typically too strong, but in the other cases, the obtained differences in binding efficiencies proved to be sufficient to enable separations from mixtures of structural analogs.

**FIGURE 6:**
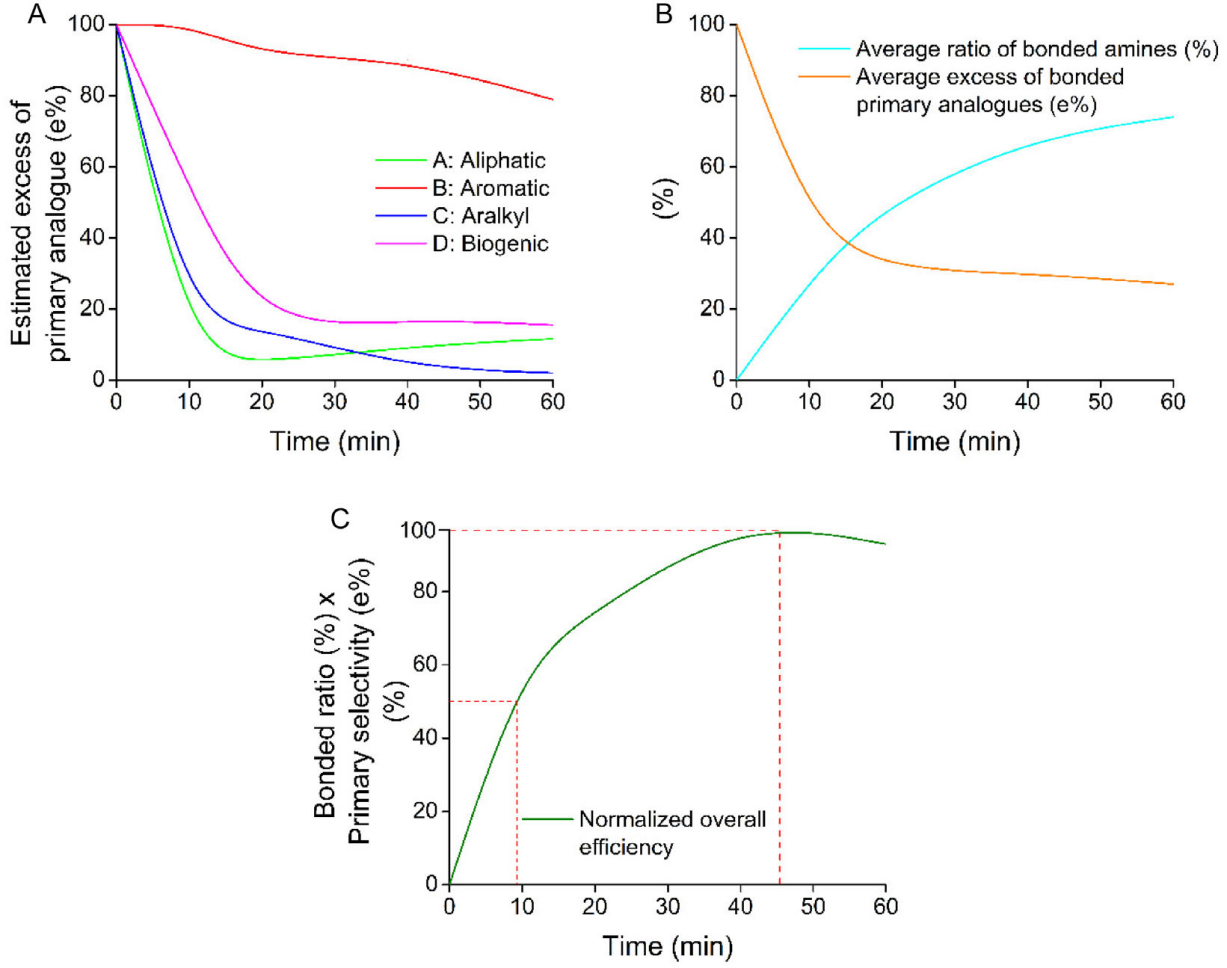
(A) Kinetic dependence of separability of primary derivatives from an equimolar mixture of primary and secondary amine analogs (estimated excess of primary derivative in different compound groups; (B) general kinetics of binding efficiency and selectivity toward primary analogs; and (C) time dependence of overall efficiency considering binding efficiency and selectivity together).

For the evaluation of the kinetic parameters, we also visualized the results as a function of the estimated excess (“theoretical excess”) of bonded primary analogs from a mixture of primary and secondary ones. Changes in separability with the progress of the process are shown in Figure 6.

The results also show that the separability (primary‐selectivity, see part “B” in Figure 6) tends to slowly decrease as we approach the equilibrium. In the equilibrium state (after 60 min), the binding sites on the surface are maximally occupied. As we could see, even the less preferred analogs have a significant affinity for the selector molecules, in spite that their complexes are less stable. The results also support that the complexation of the protonated amines with a lower degree of *N*‐substitution is not only thermodynamically, but also kinetically more stable. As we aim to exploit the differences of these complex stabilities, it seems to be advantageous to interrupt the process when the rate of complexation is lower.

Binding efficiency and selectivity were evaluated jointly as the product of their respective values (see part “C” in Figure 6), allowing determination of the optimal incubation time at 45 min. Notably, however, ≈50% of the maximum efficiency is already reached within just 10 min, which offers a practical advantage in time‐sensitive applications. The nearly identical kinetic profiles observed in different compound groups are particularly valuable, as they ensure uniform separation performance for all 96 structurally related analogs processed simultaneously on a single microplate.

### 2.4 Validation of the Method by using Mixture of Primary and Secondary Amines

To verify the practical applicability of the method, the efficiency of the separation was carried out under competitive conditions, when protonated primary‐ and secondary‐amines are simultaneously present in equimolar amounts. The efficiency of separation was determined by comparing the integrated areas of two characteristic ^1^H‐NMR peaks (see Supporting Information). The results are summarized in Table 1 and compared with those of the theoretical excess values and thermodynamic complex stability differences.

**TABLE 1:** Experimental results of the separations of multicomponent samples and the key characteristics of the theoretical separability (first values refer to 5 min incubation time, while the 60 min values refer to separation achieved at equilibrium).

1:1 Mixture of model compounds	Measured ratio of secondary derivative in supernatant (%)	Measured excess of secondary derivative in supernatant (e%)^a^	Theoretical excess of secondary derivative in supernatant (e%)^a^	Complex stability in favor of the primary derivative
**6 **+ **7**	5 min: 84% 60 min: 57%	5 min: 68e% 60 min: 14e%	5 min: 7 ± 5e% 60 min: 11 ± 5e%	four‐fold (Δlog*K* = 0.6)
**9 **+ **10**	5 min: 100% 60 min: 93%	5 min: 100e% 60 min: 86e%	5 min: 100e% 60 min: 79 ± 5e%	>10.000‐fold (Δlog*K* > 4.0)
**12 **+ **13**	5 min: 76% 60 min: 59%	5 min: 52e% 60 min: 18e%	5 min: 15 ± 5e% 60 min: 4 ± 5e%	six‐fold (Δlog*K* = 0.8)
**15 **+ **16**	5 min: 86% 60 min: 61%	5 min: 72e% 60 min: 22e%	5 min: 54 ± 5e% 60 min: 16 ± 5e%	two‐fold (Δlog*K* = 0.3)

a The e% is the excess percentage ratio, calculated according to formula reported in Subsection 4.3.

In general, studies proved a statistically significant enrichment of the initial mixtures in secondary amines for all of the model systems, since these analogs were less preferred by the immobilized selector molecules during the complexation. Experimental data revealed that in addition to thermodynamic selectivity differences, kinetic factors also significantly influence the separation outcome. In several cases, the experimentally observed efficiency order deviated from that predicted solely by the ratio of complex stability constants, indicating that binding kinetics can override thermodynamic preferences under practical conditions. (However, it has to be noted that these Δlog*K* values were determined by an indirect optochemical method for dissolved, nonimmobilized selector molecules). On the other hand, the superior performance in the case of aromatic derivatives was clearly supported by the theoretical characteristics. The observed improvement in separation at the initial stage of the process also indicates the presence of kinetics‐related factors, as already predicted by the previous studies on separability.

Moreover, the observed enrichment of secondary amine derivatives exceeds the level predicted by single‐component binding kinetics. This discrepancy arises from competitive binding, which–unusually–has a beneficial effect in this system. Since all positively charged analogs are more stabilized when bound to the selector compared to their free state, the reduced ratio of available binding sites to guest molecules under competitive conditions enhances the discrimination based on the degree of substitution.

Kinetic binding studies on single‐component systems correlated well with the experimental outcomes obtained under competitive equilibrium conditions, but proved to have weak predictive value for separation in nonequilibrium states.

In summary, all of the separations were appreciable even under adverse conditions (i.e., highly competitive complexation attributed to the excess of selector molecules, equimolar ratio of competing analogs, established equilibrium state, relatively small differences in complex stabilities, etc.). Herein, we just aimed to demonstrate the applicability under practically relevant, but not the most advantageous conditions. If the available data and the uniformity of the crude compound libraries allow an improved specification of the process, its efficiency can be highly enhanced by optimization.

**FIGURE 7:**
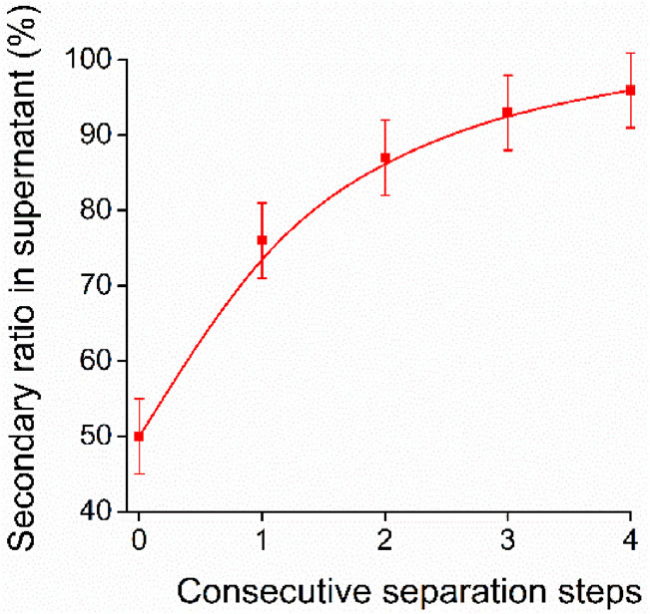
Consecutive separation from an equimolar mixture of amines **12** and **13**.

Once the first separation step is completed, further ones can also be integrated into a consecutive cascade process for an improved efficiency of purification (Figure 7).

Studies confirmed that four subsequent separation steps should be enough to reach a full isolation (>95%) of the product with the desired degree of substitution, even in the case of the weakest selectivity.

### 2.5 Reusability Study

**FIGURE 8:**
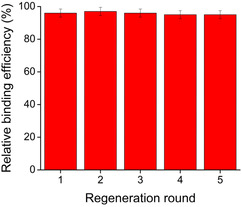
Studies on reusability of the microplate by consecutive binding of amine **9**.

We aimed to provide separation efficiency data after multiple regeneration cycles to evaluate the long‐term stability and cost‐effectiveness of the proposed method. Five consecutive binding experiments were performed on amine **9**, which showed the highest affinity to the functionalized surface among the model compounds. Results are shown in Figure 8.

The regeneration could be easily performed by fully suctioning the wells and performing five repeated extractions with methanol. The decrease in relative binding affinity is apparently negligible even after five regeneration cycles, which supports the high decomplexation efficiency of the applied liquid extraction method and the multiple reusability of the microplate device.

## 3 Conclusion

We developed a microplate‐based separation platform, which offers a transformative, automation‐ready solution for high‐throughput purification in *N*‐alkylation‐driven library syntheses. By utilizing the substitution‐dependent binding of a crown ether selector, the method enables rapid, parallel discrimination of amine analogs at the submicromolar scale ‐without the need for derivatization, crude purification, or compound‐specific method development. This game‐changer approach integrates seamlessly into automated synthesis workflows and opens new avenues for high‐throughput separations in scaffold‐synthesis‐support.

The method proved to be universally applicable for a wide range of protonated amines‐including alkyl‐, aryl‐, and aralkyl‐types‐covering compound classes of high biological and pharmaceutical relevance. Practical studies confirmed that competitive binding has a little beneficial effect on separation efficiency. A single step yields > 75% purity for alkyl and aralkyl amines, and near‐complete isolation for arylamines, within less than 10 min. This means an analytical capacity of 96 compounds per 10 min per microplate, enabling the purification of over 100,000 analogs per day using a standard liquid handling robot with plate‐to‐plate transfer compatibility. The platform is especially effective for isolating primary amines from differently substituted analogs, while the separations of secondary and tertiary ones can be achieved by fine‐tuning. Separation efficiency can be further enhanced through consecutive runs, and the microplates are easily regenerated by simple solvent extraction‐ensuring long‐term reusability.

## 4 Experimental Section

### 4.1 General

Starting materials and reagents were purchased from Sigma–Aldrich Corporation (USA, owned by Merck KGaA, Darmstadt, Germany) and used without further purification unless otherwise noted. All experiments were carried out at 25°C. Each reported data point represents the average of three parallel measurements.

All microplate‐based experiments were performed using Corning 96 Well Clear Polystyrene Microplates, nonsterile, flat‐bottom design, with a maximum well volume of 350 μL (Corning Inc., Corning, NY, USA). Automated liquid handling, microplate processing, and experimental workflows were conducted using a Biomek FXP Laboratory Automation Workstation (serial number: A318440160) (Beckman Colter Inc., Brea, CA, USA). For controlled incubation, a Heidolph Plug & Play Titramax platform shaker (Heidolph Instruments GmbH & Co. KG, Schwabach, Germany) was used.

Infrared spectra were recorded on a PerkinElmer Spectrum Two FTIR spectrometer equipped with a universal ATR accessory (PerkinElmer Inc., Waltham, MA, USA). The ATR crystal provided a penetration depth of 0–5 μm.

UV–Vis spectra were obtained using a UNICAM UV4−100 spectrophotometer controlled by VISION 3.4 software (ATI UNICAM, Knutsford, UK). Absorbance values were processed using calibration curves constructed according to the Lambert–Beer law. Standard quartz cuvettes with a 1 cm optical path length were used for all measurements.

^1^H‐NMR (300 MHz) spectra were recorded in D_2_O/MeOD using a Bruker Avance 300 spectrometer (Bruker Corporation, Billerica, MA, USA).

All data processing and visualization were carried out using OriginPro 2018 software (OriginLab Corporation, Northampton, MA, USA).

### 4.2 Surface Functionalization of the PS Microplates

In the first step, the PS surface of the microplate wells was chemically modified with chlorosulphonyl groups. Freshly distilled chlorosulphonic acid (20 µL) was added to each well of the microplate at −20°C under argon. The reaction was carried out in a vacuum desiccator by using a bed of dry ice, while the temperature of the plate surface was estimated with that of the internal atmosphere. This temperature was maintained for 30 min, during which time a bidirectional shaking (50 rpm) was also applied by placing the vacuum desiccator onto a digital shaker bed. Then concentrated sulfuric acid (98 V/V%, 3 × 150 µL/well) was added to wash out the reagent at the same temperature. Cooling is especially important in this case as the aqueous quenching of the reagent would cause the degradation of the surface due to the highly exotherm nature of the reaction. After that, the temperature was raised to 0°C and each well was washed further with 3 × 150 µL of distilled water to remove the acid residues.

In the next step, 3‐bromopropylamine hydrobromide (8.0 M solution in 20 w/w% trimethylamine/water; 50 µL/well) as a bifunctional linker was added to each well under argon at 20 °C. The system was heated to 50 °C by using an external infrared lamp, while the plate was continuously shaken during the 8 h‐treatment. The wells were washed with distilled water (3 × 150 µL), then with methanol (10 × 150 µL). As a result, the amino groups were anchored by forming sulfonamide bonds, while the terminal bromide of the linker can be used to immobilize the selector molecules.

For the covalent attachment of the selector molecules as the last step of the functionalization, the *OH*‐functionalized macrocycle was dissolved in acetonitrile (this is compatible with the PS plate, can dissolve the macrocycle and can be homogeneously mixed with the solution of the base) and this solution (50 µL/well, 1 mg macrocycle dissolved in 10 µL acetonitrile) was added to each well followed by the addition of 20 w/w% KOH in water (20 µL/well to activate the aromatic *OH* group of the selector molecule by deprotonation). The plate was then treated for 8 h while shaking at 50 °C under argon. The wells were finally washed with distilled water (3 × 150 µL/well) and methanol (3 × 150 µL/well) and dried at 20 °C under argon. The functionalized microplates were stored in air at 20 °C until use.

### 4.3 Studies on Separability

Studies on separability were carried out by applying 300 µL of 10^−3^ M single‐component aqueous solutions of the model amines in the microplate wells. Liquid handling was performed with multichannel pipettes. After pipetting the initial solution into the microplate wells, the microplate was incubated while applying continuous shaking (50 rpm) on a shaker plate. In‐process sampling was carried out by using Hamilton‐syringe (10 µL), while the solution phase was removed by pipetting at the end of the separation. Only the upper 150 µL of the solution in each well was used for purification. The remaining 150 µL of the initial amine solution was not investigated in separation (it can be purified in a subsequent step) due to the discussed limitations of the pipetting‐based concept in liquid handling. The concentrations of the aqueous solutions were determined spectroscopically, relying upon a UV/V is calibration curve based on the Lambert–Beer equation (detailed information can be found in the Supporting Information) after a proper dilution with distilled water. These concentrations were then used to calculate the relative amounts of the remaining dissolved amine and the results were expressed as the ratio of the bonded ones by subtracting the dissolved ones from the initial full amounts. The deviations of the reported points are within ±5% in each case. The regeneration of the wells between two measurements was performed by a simple washing procedure by adding 5 × 300 µL methanol and 3 × 300 µL of distilled water, subsequently removing the full amount of the solutions by pipetting. For separation studies, we also used e% as an output parameter, which was calculated according to the following formula

(1) $$e\%=\frac{R_{\mathrm{prim}}\left(\%\right)-R_{\sec}(\%)}{R_{\mathrm{prim}}(\%)+R_{\sec}(\%)}\cdot 100$$

where *R*_prim_ is the percentage ratio of the bonded primary analog in single‐component samples at a given incubation time, while *R*_sec_ is that for the bonded secondary derivative. These e% values were applied for single‐component samples to predict separability and for multicomponent mixtures to evaluate the separation efficiency.

### 4.4 Validation of the Method

Measurements, including initialization, incubation, and sampling were carried out in the same manner as described for studies on separability in Subsection 4.3. The only difference is that the source phase was a two‐component equimolar mixture of primary and secondary amines with a total concentration of 2 × 10^−3 ^M in a pH = 7.0 aqueous solution. After the procedure starting from the 1:1 mixture was completed, the resulted aqueous samples, which were enriched in the less preferred (i.e., more weakly bonded secondary amine derivatives) analog, were evaporated under reduced pressure. The solid residues were dried, dissolved in MeOD/D_2_O and forwarded to ^1^H‐NMR measurements. The efficiency of the enrichment in the less preferred analogs was evaluated based on the relative molar ratio of that in the solution phase after the incubation. This was calculated based on the comparison of the integrals of two characteristic ^1^H‐NMR‐peaks of the analogs in each case, which ratio is directly correlated with the ratio of the corresponding derivatives in the sample. The corresponding spectra can be found in the Supporting Information. The applied incubation time was uniformly 5 min, while the deviation of the results was within ±5.0% in all cases.

## Supporting Information

Additional supporting information can be found online in the Supporting Information section. **Supporting** **Fig.** **S1** **:** UV‐absorption spectrum of the applied selector molecule (**4**). **Supporting Fig. S2** **:** Spectrum for alkylamine mixture in MeOD. **Supporting Fig. S3** **:** Isolated spectrum of amine **6** in MeOD. **Supporting Fig. S4:** Isolated spectrum of amine **7** in MeOD. **Supporting** **Fig. S5** **:** Spectrum for aromatic amine mixture in MeOD. **Supporting** **Fig. S6** **:** Spectrum for aralkylamine mixture in MeOD. **Supporting** **Fig. S7:** Spectrum for biogenic amine mixture in MeOD. **Supporting** **Table S1:** UV‐based calibration parameters for model amines.

## Author Contributions

**Panna Vezse:** investigation, formal analysis, visualization, writing – original draft. **Tünde Tóth:** writing – review and editing, resources. **Péter Huszthy:** writing – review and editing, resources. **Ádám Golcs:** conceptualization, methodology, investigation, writing – original draft, supervision, project administration.

## Conflicts of Interest

The authors declare no conflicts of interest.

## Supporting information

Supplementary Material

## Data Availability

The authors confirm that the data supporting the findings of this study are available within the article and its Supporting Information.
