# Supplementary material for: Multiple‐Use Microplate Assay for Submicromolar Ultra High‐Throughput Separation of Amines Based on their Degree of Substitution
Source: ChemistryOpen. 2025 Dec 1;15(4):e202500491. doi: 10.1002/open.202500491 (PMC13052240; doi:10.1002/open.202500491)
Supplement: Supplementary file 1 — Supplementary Material [file OPEN-15-e202500491-s001.pdf]

Supporting Information

Multiple-Use Microplate Assay for Submicromolar Ultra High-Throughput Separation of Amines Based on Their Degree of Substitution

Panna Vezse, [a] Tünde Tóth, [a,b] Péter Huszthy [a] and Ádám Golcs *[a,c,d]

[a] P. Vezse, Dr. T. Tóth, Prof. Dr. P. Huszthy, Dr. Á. Golcs
Department of Organic Chemistry and Technology, Faculty of Chemical Technology and Biotechnology
Budapest University of Technology and Economics
Műegyetem rkp. 3., H-1111 Budapest, Hungary
E-mail: golcs.adam@semmelweis.hu

[b] Dr. T. Tóth
HUN-REN Centre for Energy Research
Konkoly-Thege Miklós út 29-33., H-1121 Budapest, Hungary

[c] Dr. Á. Golcs
Department of Pharmaceutical Chemistry
Semmelweis University
Hőgyes Endre u. 9., H-1092 Budapest, Hungary

[d] Dr. Á. Golcs
Center for Pharmacology and Drug Research & Development
Semmelweis University
Üllői u. 26., H-1085, Budapest, Hungary

Content

1. **UV-absorbance-based determination of the loading density of macrocycle 4**
2. **UV-absorbance-based determination of the concentration of model amines**
3. **^{1}H-NMR-based determination of the supernatant composition after separation**

1. UV-absorbance-based determination of the loading density of macrocycle 4

In order to give information on the extent of labeling by the immobilized selector molecule, the remaining amount of macrocycle **4** was determined using UV-absorbance spectroscopy after the immobilization procedure. In the last step of the procedure, the ratio of unbound selector molecules was calculated relying on a UV/Vis calibration curve based on the *Lambert–Beer* equation. The calibration was applied at 263 nm absorption peak-wavelength (**Figure S1**) using 10^{-5} - 10^{-6} M acetonitrile solutions of macrocycle **4**.

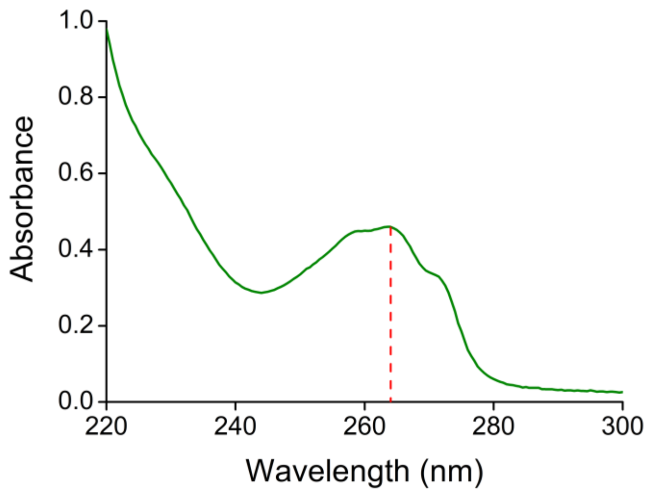

Figure S1. UV-absorption spectrum of the applied selector molecule (**4**)

The samples of the macrocycle solutions were diluted to the concentration gradient of the calibration, then the loading density of the selector molecule was subsequently determined indirectly from the concentration of the initial solution used for the surface treatment. Based on this calculation, 7±2% of the applied macrocycles were covalently immobilized to the surface.

2. UV-absorbance-based determination of the concentration of model amines

Table S1. UV-based calibration parameters for model amines

Amine	$\lambda_{calibration}$ (nm)	Calibration concentration range (M)	Equation of the calibration curve	R^2
**9**	230	$1\cdot10^{-5}$-$1\cdot10^{-4}$	A=2284·c+0.053	0.999
**10**	238	$1\cdot10^{-5}$-$1\cdot10^{-4}$	A=3586·c+0.000	0.999
**11**	245	$1\cdot10^{-5}$-$1\cdot10^{-4}$	A=1374·c+0.007	0.998
**12**	256	$1\cdot10^{-5}$-$1\cdot10^{-4}$	A=947·c+0.000	0.998
**13**	210	$1\cdot10^{-5}$-$1\cdot10^{-4}$	A=1518·c+0.000	0.999
**14**	262	$1\cdot10^{-5}$-$1\cdot10^{-4}$	A=557·c+0.000	0.998
**6**	210	$5\cdot10^{-5}$-$1\cdot10^{-4}$	A=2090·c-0.105	0.999
**7**	200	$5\cdot10^{-5}$-$1\cdot10^{-4}$	A=1804·c-0.090	0.998
**8**	200	$5\cdot10^{-5}$-$1\cdot10^{-4}$	A=368·c-0.018	0.998
**17**	275	$5\cdot10^{-6}$-$5\cdot10^{-5}$	A=1440·c+0.004	0.999
**15**	275	$5\cdot10^{-6}$-$5\cdot10^{-5}$	A=848·c+0.002	0.998
**16**	275	$5\cdot10^{-6}$-$5\cdot10^{-5}$	A=874·c+0.003	0.998
**18**	256	$5\cdot10^{-6}$-$5\cdot10^{-5}$	A=1045·c-0.012	0.998

3. ^{1}H-NMR-based determination of the supernatant composition after separation

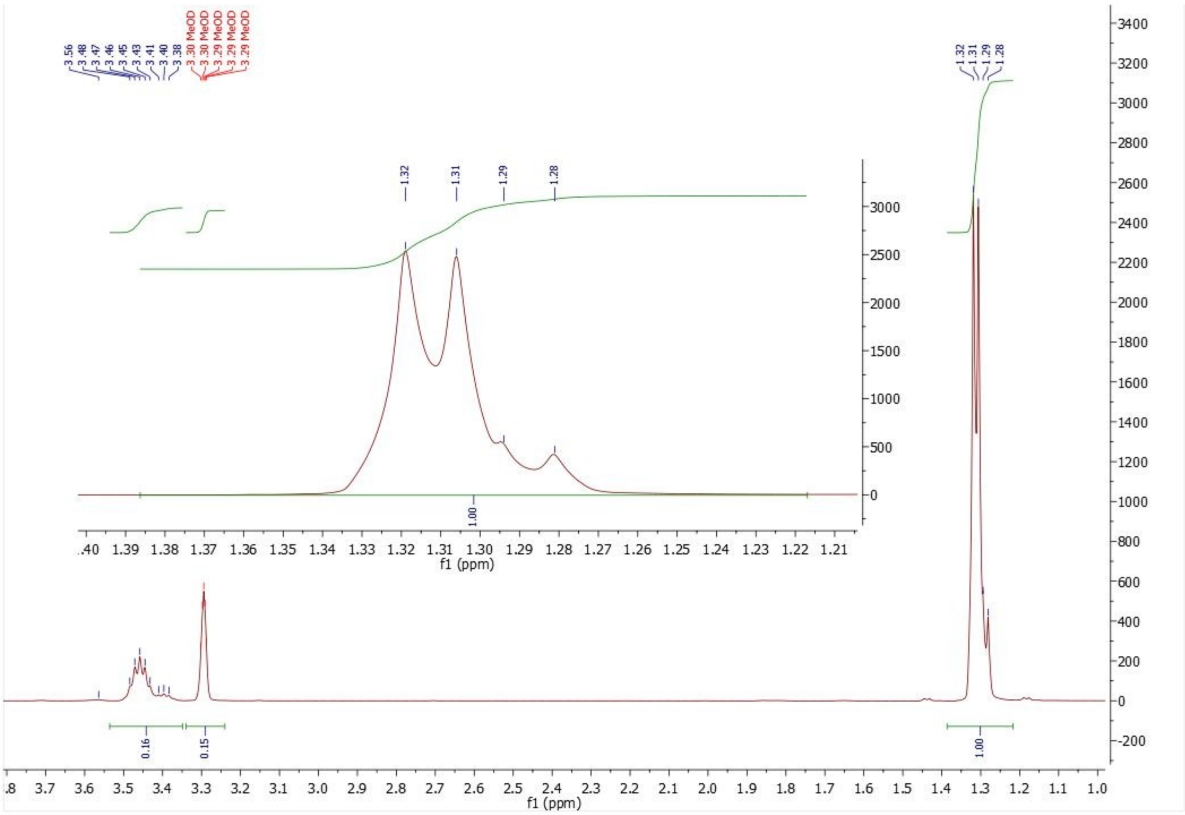

Figure S2. Spectrum for alkylamine mixture in MeOD

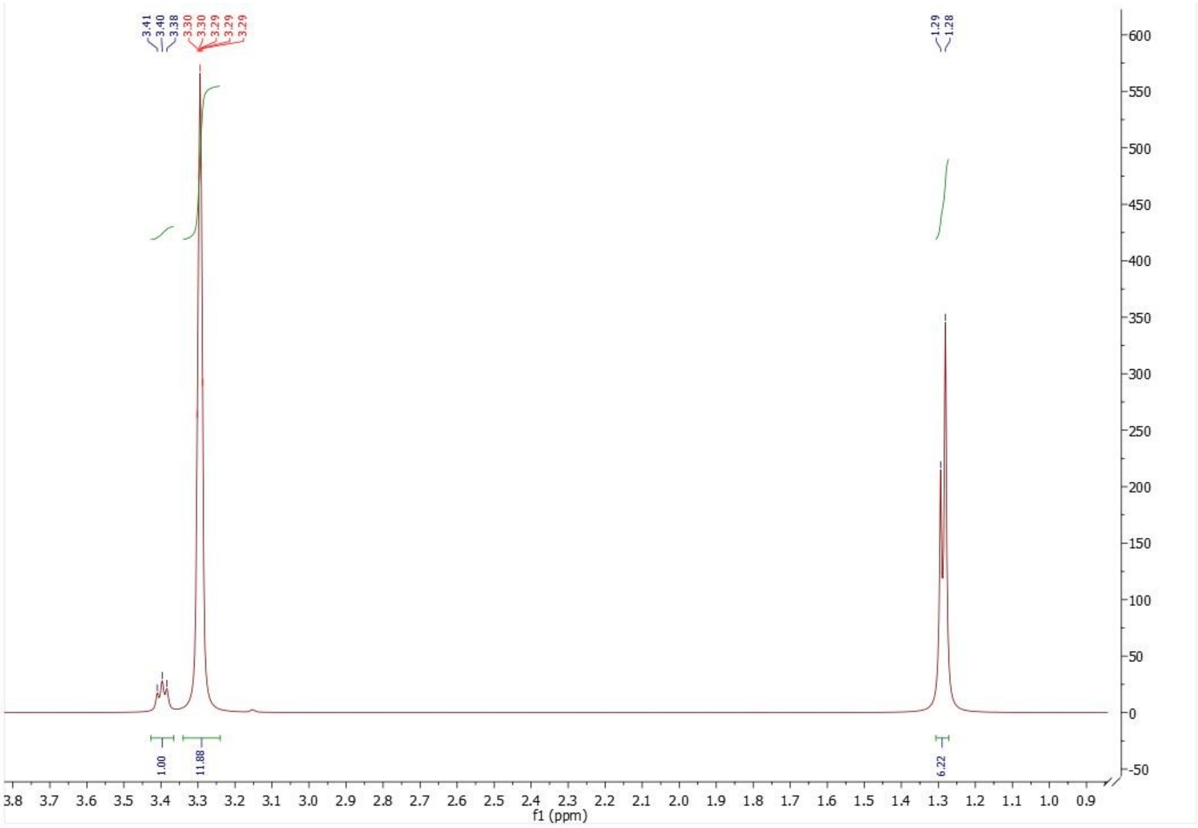

Figure S3. Isolated spectrum of amine **6** in MeOD

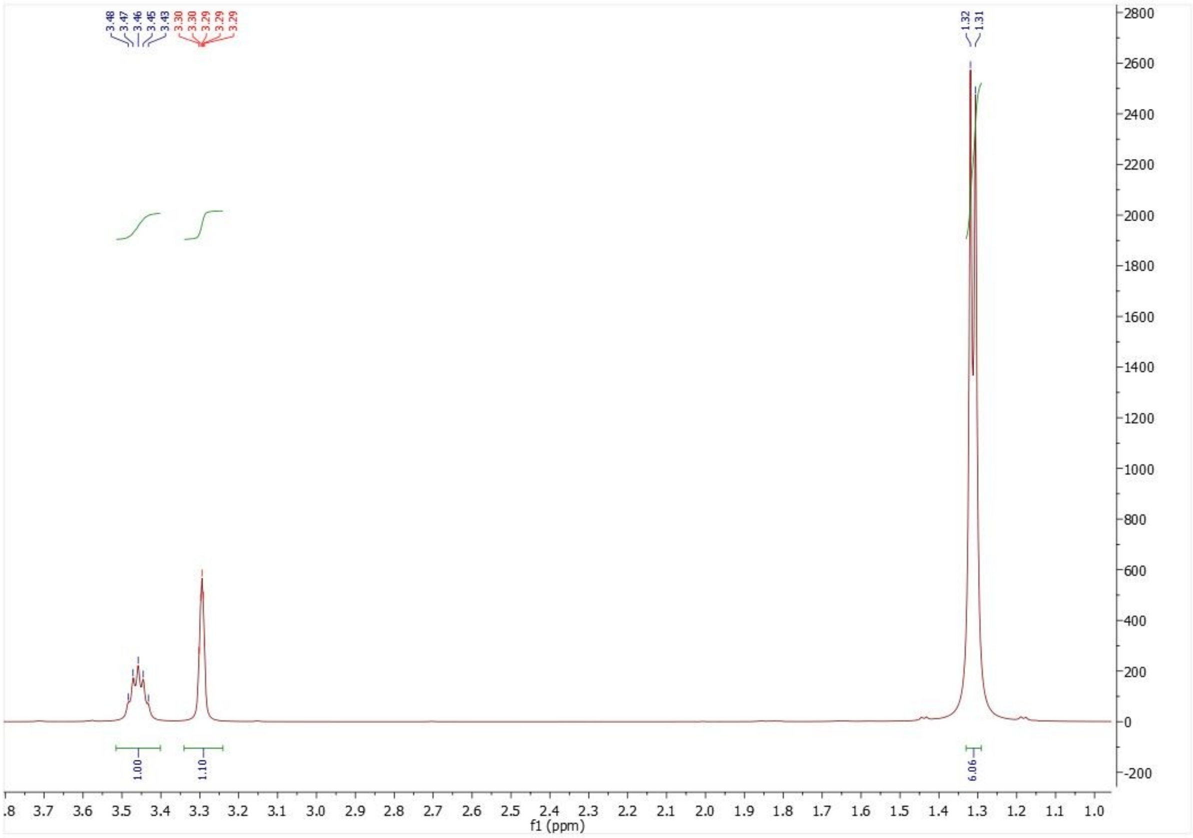

Figure S4. Isolated spectrum of amine **7** in MeOD

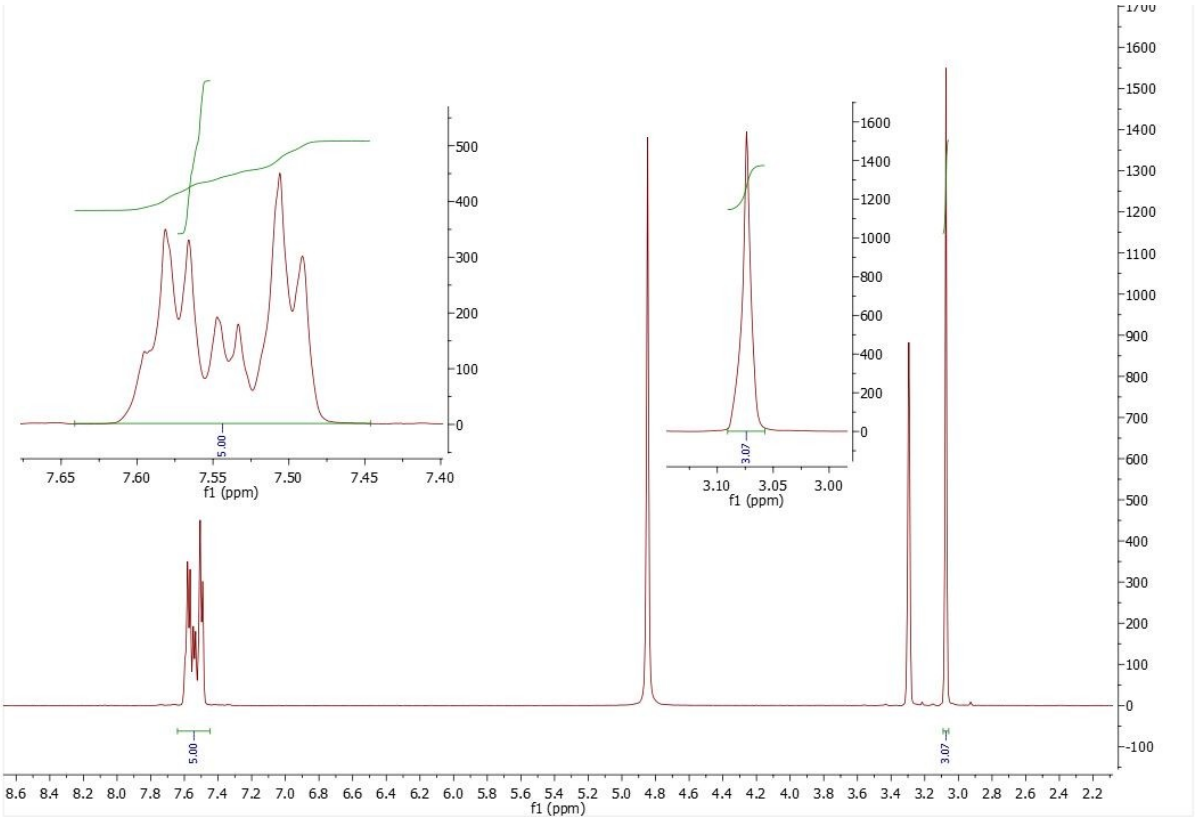

Figure S5. Spectrum for aromatic amine mixture in MeOD

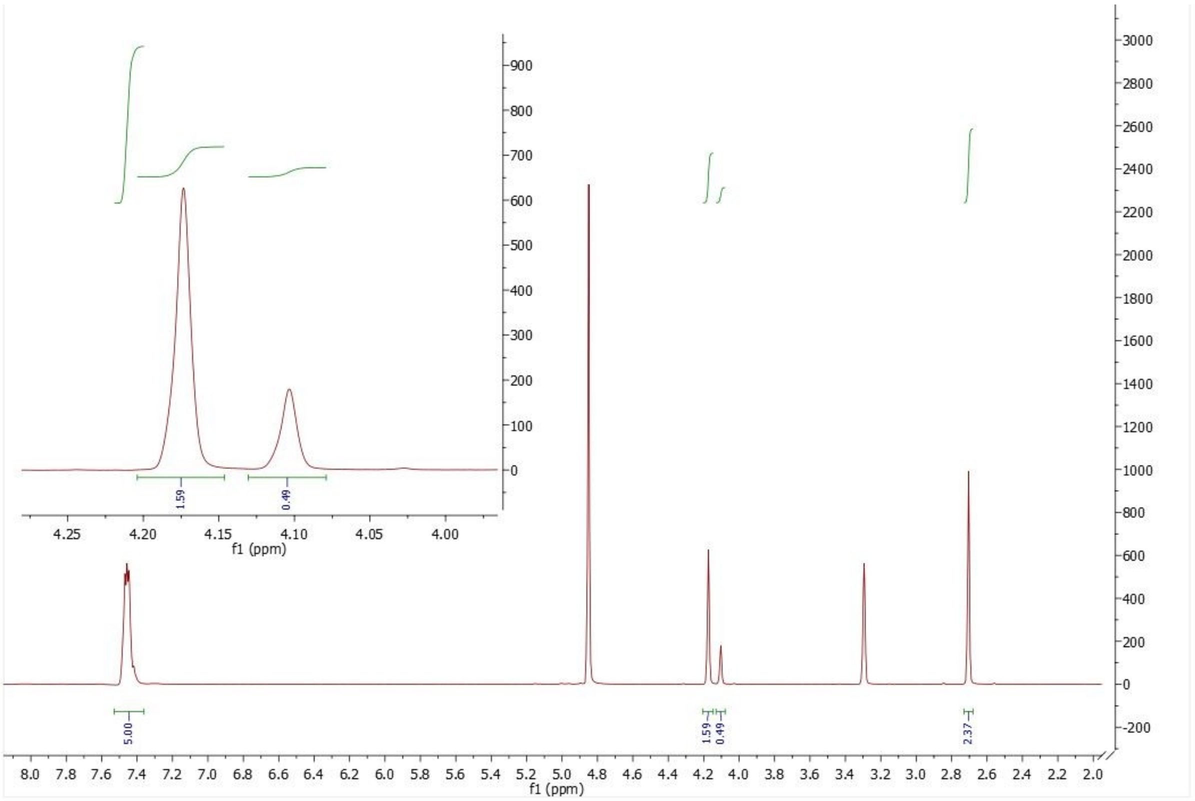

Figure S6. Spectrum for aralkylamine mixture in MeOD

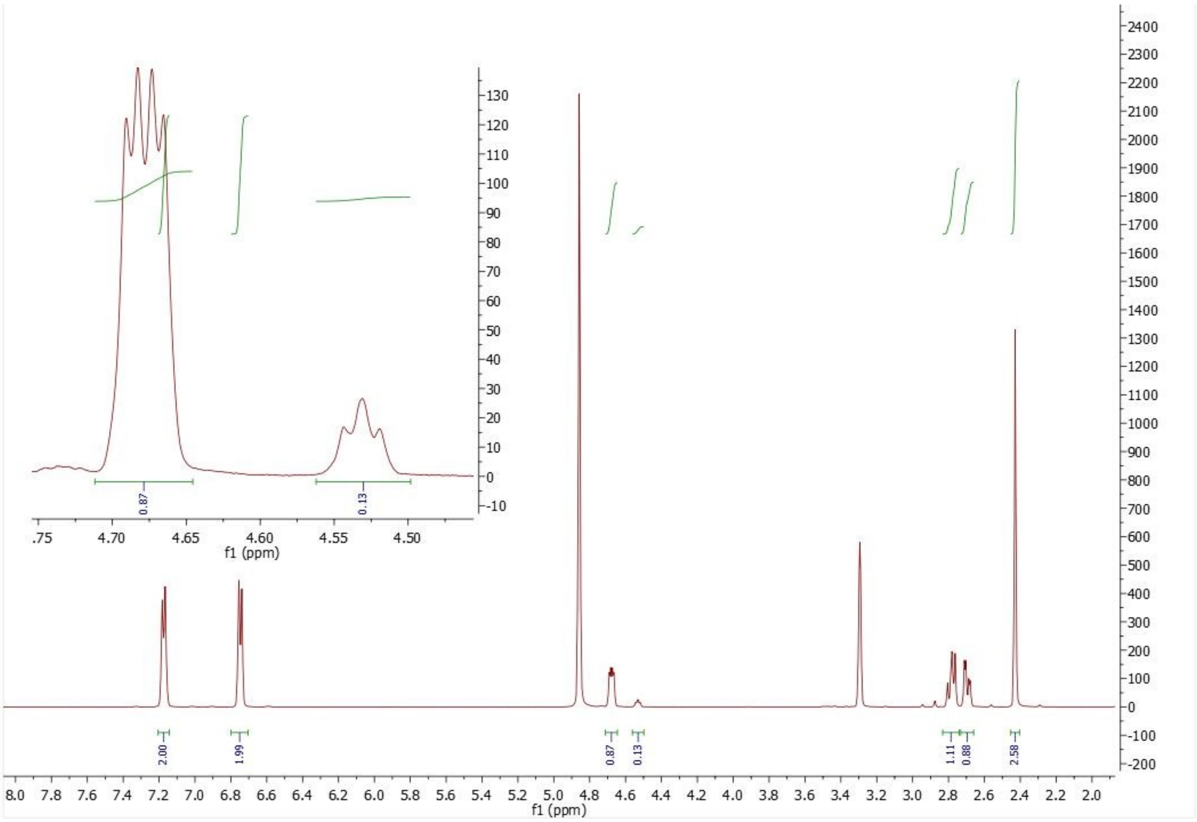

Figure S7. Spectrum for biogenic amine mixture in MeOD
